# Glucocorticoids and Stress-Induced Changes in the Expression of PERIOD1 in the Rat Forebrain

**DOI:** 10.1371/journal.pone.0130085

**Published:** 2015-06-15

**Authors:** Sherin Al-Safadi, Marie Branchaud, Spencer Rutherford, Shimon Amir

**Affiliations:** 1 Department of Biology, Concordia University, SP-375.07, 7141 Sherbrooke Street West, Montréal, Quebéc, H4B 1R6, Canada; 2 Department of Psychology, Center for Studies in Behavioral Neurobiology, Concordia University, SP-244, 7141 Sherbrooke Street West, Montréal, Quebéc, H4B 1R6, Canada; University of Lübeck, GERMANY

## Abstract

The secretion of glucocorticoids in mammals is under circadian control, but glucocorticoids themselves are also implicated in modulating circadian clock gene expression. We have shown that the expression of the circadian clock protein PER1 in the forebrain is modulated by stress, and that this effect is associated with changes in plasma corticosterone levels, suggesting a possible role for glucocorticoids in the mediation of stress-induced changes in the expression of PER1 in the brain. To study this, we assessed the effects of adrenalectomy and of pretreatment with the glucocorticoid receptor antagonist, mifepristone, on the expression of PER1 in select limbic and hypothalamic regions following acute exposure to a neurogenic stressor, restraint, or a systemic stressor, 2-Deoxy-D-glucose (2DG) in rats. Acute restraint suppressed PER1 expression in the oval nucleus of the bed nucleus of the stria terminalis (BNSTov) and the central nucleus of the amygdala (CEAl), whereas 2DG increased PER1 in both regions. Both stressors increased PER1 expression in the paraventricular (PVN) and dorsomedial (DMH) nuclei of the hypothalamus, and the piriform cortex (Pi). Adrenalectomy and pretreatment with mifepristone reversed the effects of both stressors on PER1 expression in the BNSTov and CEAl, and blocked their effects in the DMH. In contrast, both treatments enhanced the effects of restraint and 2DG on PER1 levels in the PVN. Stress-induced PER1 expression in the Pi was unaffected by either treatment. PER1 expression in the suprachiasmatic nucleus, the master circadian clock, was not altered by either exposure to stress or by the glucocorticoid manipulations. Together, the results demonstrate a key role for glucocorticoid signaling in stress-induced changes in PER1 expression in the brain.

## Introduction

The circadian clock drives daily rhythms in behavior and physiology and these rhythms help animals adapt to their cyclic environment. In mammals, circadian rhythms depend on the coordination of the master clock in the suprachiasmatic nucleus (SCN) with subordinate circadian clocks elsewhere in the brain and in the periphery [1, 2]. This coordination is sensitive to a range of external and systemic stressful conditions that disrupt homeostasis, leading to dysregulation of circadian physiological and behavioral rhythms [3–5]. Studies in rodents suggest that the effect of stress is independent of the SCN clock and point to changes in the expression of clock genes elsewhere in the brain and periphery as a possible mechanism [6–8]. How stress might affect clock gene expression in the brain is not fully understood, but several lines of evidence suggest that glucocorticoid hormones, primary effectors of the neuroendocrine stress system, might play a role [7, 9, 10]. For example, it has been shown that treatment with dexamethasone, a synthetic glucocorticoid, induces circadian gene expression in cultured rat-1 fibroblasts, and can transiently alter the phase of circadian gene expression in the liver, kidneys, and heart [11, 12]. Furthermore, we have shown that depletion of endogenous glucocorticoids via adrenalectomy (ADX) or selective genetic deletion of brain glucocorticoid receptors (GR), blunt the rhythm of expression of the clock protein, PER2 in nuclei of the central extended amygdala, the oval nucleus of the bed nucleus of the stria terminalis (BNSTov) and the lateral part of the central nucleus of the amygdala (CEAl), without affecting the rhythms of the SCN in rats [13–16].

The clock protein PER1 is a core component of the circadian oscillator [1, 17]. It is expressed in the SCN, as well as in many other central structures that play diverse roles in behavior and physiology including structures important in stress, autonomic control and emotion regulation [8, 18–20]. In recent work, we and others have shown that stressors known to induce the release of the adrenal glucocorticoid hormone, corticosterone (CORT), rapidly alter the expression of PER1 in various hypothalamic and limbic nuclei and in some peripheral tissues such as the liver, but not in the SCN [8, 9, 18, 20–24]. Furthermore, it has been shown that the effect of stress on *Per1* expression in the liver is mediated by a glucocorticoid-responsive element (GRE) present on the promoter region of *Per1*, directly implicating glucocorticoids and their receptors in the stress-induced modulation of PER1 expression in the periphery [9].

In the present study, we investigated the involvement of endogenous glucocorticoids and GR in the rapid changes in PER1 expression induced by acute exposure to two qualitatively different stressors, restraint and 2-Deoxy-D-glucose (2DG) administration. While the former stressor activates the HPA axis indirectly (through corticolimbic pathways), eliciting acute emotional behavior, the latter represents a direct and invasive challenge that disrupts internal homeostasis without an affective response [25, 26]. We found previously that these two different types of stress, restraint and 2DG, exert opposite effects, decreases and increases, respectively, on PER1 expression in the BNSTov and CEAl while exerting similar effects in other forebrain regions, including the paraventricular nucleus of the hypothalamus (PVN), dorsomedial hypothalamus (DMH), and piriform cortex (Pi) in rats [21]. Here, we found that depletion of endogenous glucocorticoids via ADX or pharmacological blockade of GR (GRX) altered the acute effect of stress on PER1 expression in the forebrain in a stress- and region-specific manner, pointing to a complex interaction between stress type, glucocorticoid signaling and PER1 in the forebrain.

## Materials and Methods

### Animals and Housing

Animal handling and housing procedures adhered to the Canadian Council on Animal Care guidelines, and were approved by the Animal Care Committee of Concordia University. Intact, ADX and sham-operated male Wistar rats weighing 125–150 g were purchased from Charles River Laboratories (St. Constant, QC, Canada). As described in detail previously [15,21], rats were housed in transparent plastic cages (24cm wide x 20.5cm height x 40cm deep) individually, under a 12:12h light (100 lux at cage bottom)/dark (LD) cycle. Each cage was kept in a temperature-controlled (22°C), ventilated, sound- and light-attenuated isolation box, and was equipped with a running wheel. Running wheel data were recorded (VitalView software, Mini-Mitter,Sunriver, OR, USA) and actograms were analyzed (Actiview Biological Rhythm Analysis software, Mini-Mitter) to ensure that rats were stably entrained to the 12:12h LD cycle. Rats were allowed free access to food and water at all times. During the 2-week entrainment period, ADX rats were given CORT in their drinking water to prevent disruption of normal PER rhythms as previously described [15, 16]. CORT (Sigma-Aldrich, Oakville, ON, Canada) was dissolved in 0.9% saline, at a concentration of 25 mg/l [15]. Sham rats were provided with 0.9% saline only. On the morning of the test day, prior to stress exposure, the drinking fluid of ADX rats was replaced with 0.9% saline only.

### Drugs

The non-selective GR antagonist mifepristone (Sigma-Aldrich, Oakville, ON, Canada) was dissolved in 0.9% saline containing 5% dimethyl sulfoxide (Sigma-Aldrich, Oakville, ON, Canada) and 1% Tween 20 (Fischer Scientific, Ottawa, ON, Canada). The suspension was vortexed 1 min prior to drug injection, and as needed throughout the dosing. The drug was administered i.p. at a concentration of 40 mg/kg. A similar dose was shown to attenuate stress-induced changes in a number of behavioral and physiological parameters in rats [27]. Control rats received vehicle only.

### Stressors

#### Restraint

Rats were exposed to a 30 min restraint challenge in custom-designed ventilated Plexiglas tubes (7 mm thick, internal diameter of 75 mm, adjustable in length from 130–180 mm). Control rats were handled only.

#### 2DG

Rats received a subcutaneous injection of 250 mg/kg 2DG (Sigma-Aldrich, Oakville, ON, Canada) in 0.9% saline. Control rats were injected with vehicle only.

### Plasma CORT Collection and Analysis

Rats were placed in restraining devices and then tail-clipped using a razor and capillary tubes (0.5 ml) for blood sampling prior to perfusion. Blood samples were centrifuged for 10 min at 13,000 rpm, 4°C, and the plasma was extracted and stored at -80°C, as previously described [16,21]. Circulating plasma CORT levels were determined using a CORT Enzyme Immunoassay kit (Enzo Life sciences, Farmingdale, NY, USA).

### Tissue Preparation and Immunohistochemistry

As described in detail previously [14,21], rats were anesthetized with sodium pentobarbital (100 mg/kg, i.p.) and perfused transcardially with 300 ml of cold 0.9% saline (4°C, 0.9% NaCl in distilled water), followed by 300 ml of cold 4% paraformaldehyde (4°C, 4% paraformaldehyde in 0.1 M phosphate buffer). Brains were gently extracted then fixed in 4% paraformaldehyde at 4°C, for approximately 24 h. A vibratome was used to slice each brain into a series of 50 μm serial coronal sections. These sections were then placed in Watson’s Cryoprotectant and stored at −20°C. PER1 immunohistochemistry was performed using an affinity-purified rabbit polyclonal antibody, raised against PER1 (1:24,000—R1177, EMD-Millipore), as previously described in depth [19,21]. Brain sections were incubated for 40 h in a primary solution that consisted of 2% normal goat serum, PER1 polyclonal rabbit antibodies, and 5% milk buffer in a Triton Trizma-buffered saline solution (0.3% Triton, 50 mM Trizma buffer, 0.9% saline), at 4°C [21]. Following, all sections were incubated in a secondary solution, composed of biotinylated anti-rabbit IgG rasied in goat (1:200, Vector Laboratories, Burlington, ON, Canada). Lastly, as described previously [19,21], the sections were incubated in a tertiary Avidin-Biotin-Peroxidase solution (Vectastain Elite ABC Kit, Vector Laboratories), thoroughly washed in a 0.5% 3,3-diaminobenzidine (DAB) solution, and then stained using a 0.5% DAB, 0.01% H_2_O_2_ and 600μL of 8% NiCl_2_ solution in 150mL.

### Microscopy and Data Analysis

DAB-stained sections were gently placed onto gel-coated slides, dehydrated in alcohol and Citrisolv (Fisher), then coverslipped and left to dry. A light microscope (Leica, DMR) and a Swanson rat brain atlas (Swanson, 2004, Brain Maps: Structure of Rat Brain) were used to identify the sections. As previously described in detail [21], images of the BNSTov, CEAl, PVN, DMH, Pi and SCN were captured with a Sony XC-77 video camera, Scion LG-3 frame grabber (Scion Corporation, Frederick, MD, USA), and Image SXM software (http://www.ImageSXM.org.uk1 v1.95, S.D. Barret). Using counts from six unilateral images showing the highest number of labeled nuclei, the mean number of PER1 immunoreactive (IR) cells per region was then calculated for each animal, as previously described [13,21]. Capturing of images and counting of cells were performed blind. Data were analyzed using a two-way analysis of variance (ANOVA), with a confidence level [α] set at 0.05. Differences between stressed and control groups were revealed using Bonferroni or Dunnett’s post-hoc analyses. In both the ADX and GRX experiments, data from control groups for restraint and 2DG were tested and determined to have no significant differences (data not shown), and accordingly, pooled together.

### Experimental Protocol

#### Adrenalectomy experiment

On the test day, the ADX and sham-operated rats (*n* = 4 per group) were exposed to restraint or given an injection of 2DG, at zeitgeber time 2 (ZT2, ZT0 indicates time of lights on). Control rats for restraint were handled only and those for 2DG were injected with vehicle. Blood was collected 1 h after stress onset (ZT3) and rats were then immediately anesthetized and perfused. The time points selected were based on our previous findings that demonstrated maximal changes in PER1 expression 1 h after exposure to stress, during the light phase of the LD cycle, a time when levels of circulating glucocorticoids are know to be at their lowest [21].

#### Mifepristone experiment

Groups of rats (*n* = 4 per group) were treated with the GR antagonist, mifepristone, or vehicle, 1 h prior to the onset of the stressors, at ZT1. Rats were then exposed to either restraint or 2DG at ZT2, and subsequently treated at ZT3 as described above.

## Results

### Adrenalectomy experiment

**Fig 1:**
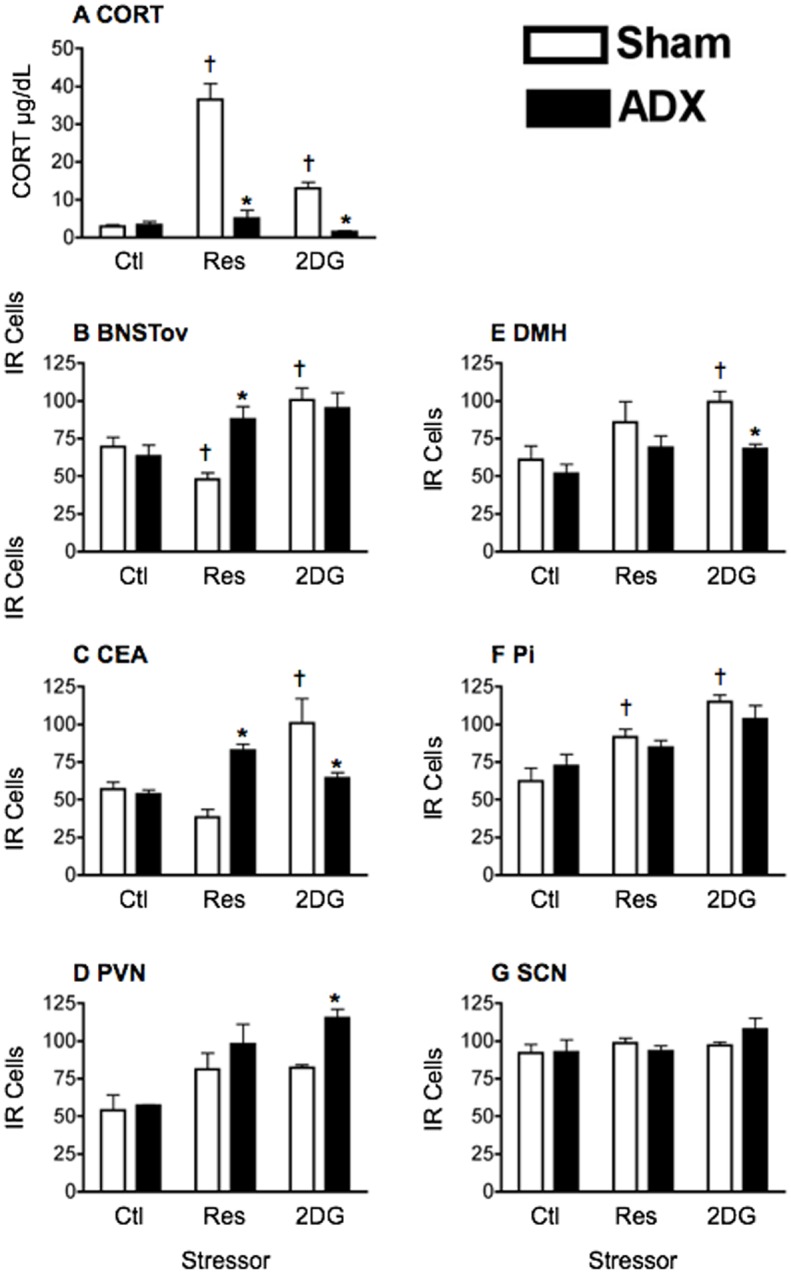
Effect of adrenalectomy on stress-induced changes in plasma CORT levels and PER1 expression. A) Plasma corticosterone (CORT) levels as a function of the category of stress and adrenalectomy. Sham-operated (sham) and adrenalectomized (ADX) rats were handled only or exposed to restraint or 2DG at ZT2. Plasma was collected 1 h later at ZT3. The bars and vertical lines represent means ± SEM, *n* = 4 per group. * Significant difference from corresponding control group (*p*<0.05). B-G) Number of PER1 immunoreactive (IR) cells in different forebrain and hypothalamic nuclei at ZT3. IR cells are shown as a function of the category of stress in sham-operated (sham) and adrenalectomized (ADX) rats. Means ± SEM are shown, *n* = 4 per group; * significant difference from corresponding sham group, *p*<0.05. significant difference from main control group, *p*<0.05.

**Fig 2:**
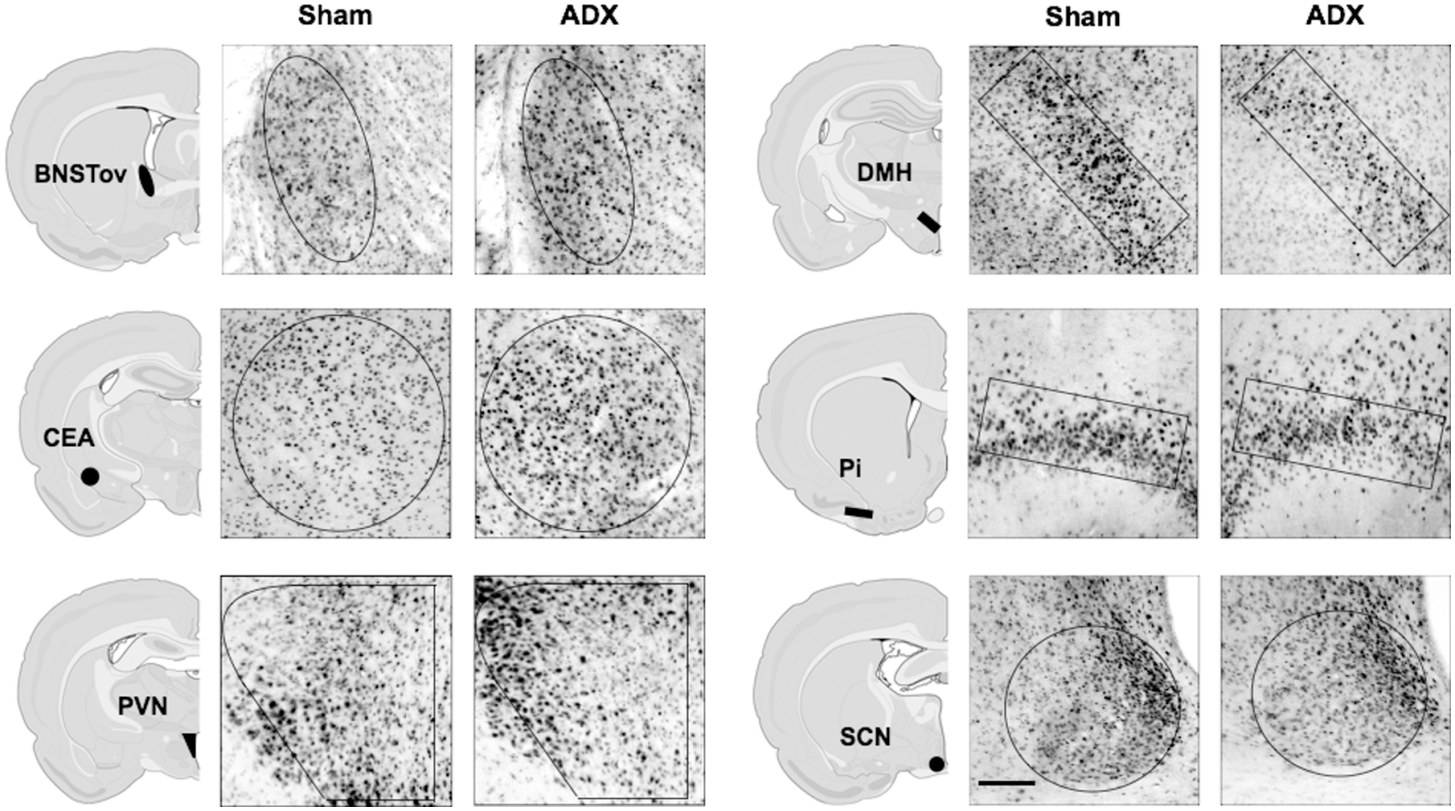
Examples of PER1 expression in different forebrain and hypothalamic nuclei of Sham and adrenalectomized rats. Sham or adrenalectomized (ADX) rats were exposed to a 30 min restraint session at ZT2, and then killed at ZT3 (scale bar: 100μm).

As expected, plasma CORT levels were elevated in sham-operated rats 1 h following acute exposure to both the restraint and 2DG stressors, with significantly higher levels seen following restraint than 2DG (main effect of stress: F_2,18_ = 37.48, *p*<0.0001; Fig 1A). These effects were absent in ADX rats (main effect of treatment: F_1,18_ = 66.8, *p*<0.0001; interaction: F_2,18_ = 28.69, *p*<0.0001; Fig 1A). The effect of ADX on the stress-induced changes in PER1 expression is shown in Fig 1B–1G, Fig 2 and statistical analyses in Table 1. As can be seen in Fig 1, in sham-operated rats, restraint stress suppressed PER1 expression in both the BNSTov and CEAl (Fig 1B and 1C) and increased PER1 expression in the PVN, DMH, Pi (Fig 1D–1F). ADX reversed the suppressive effect of restraint in the BNSTov and CEAl (Fig 1B and 1C), whereas in contrast, the stimulatory effect of restraint stress on PER1 expression in the PVN, DMH and Pi was not significantly attenuated (Fig 1D–1F). Treatment with the systemic stressor 2DG increased PER1 levels in all regions studied including the BNSTov and CEAl, but not in the SCN (Fig 1B–1G). ADX reversed this increase in the CEAl and DMH (Fig 1C and 1E). Contrastingly however, ADX significantly enhanced the stimulatory effect of 2DG on PER1 levels in the PVN (Fig 1D). The 2DG-induced increase in PER1 expression in the BNSTov and Pi was unaffected by ADX (Fig 1B and 1F). PER1 expression in the SCN was not affected by either stressor or by ADX (Fig 1G).

**Table 1:** Statistical analysis (ANOVA) of the effect of adrenalectomy (ADX) and stress type (Stressor) on PER1 expression in limbic and hypothalamic brain regions.

Structure	ADX	Stressor	ADX x Stressor
BNSTov	F(1,18) = 2.26, ns	F(2,18) = 10.75, *p*<0.001	F(2,18) = 5.99, *p*<0.05
CEA	F(1,18) = 0.06, ns	F(2,18) = 7.03, *p*<0.01	F(2,18) = 13.78, *p*<0.001
PVN	F(1,18) = 6.41, *p*<0.05	F(2,18) = 14.37, *p*<0.001	F(2,18) = 1.57, ns
DMH	F(1,18) = 8.84, *p*<0.01	F(2,18) = 5.0, *p*<0.05	F(2,18) = 1.34, ns
Pi	F(1,18) = 0.24, ns	F(2,18) = 18.03, *p*<0.0001	F(2,18) = 1.33, ns
SCN	F(1,15) = 0.17, ns	F(2,15) = 1.4, ns	F(2,15) = 0.86, ns

*ns* not significant

### Mifepristone experiment

**Fig 3:**
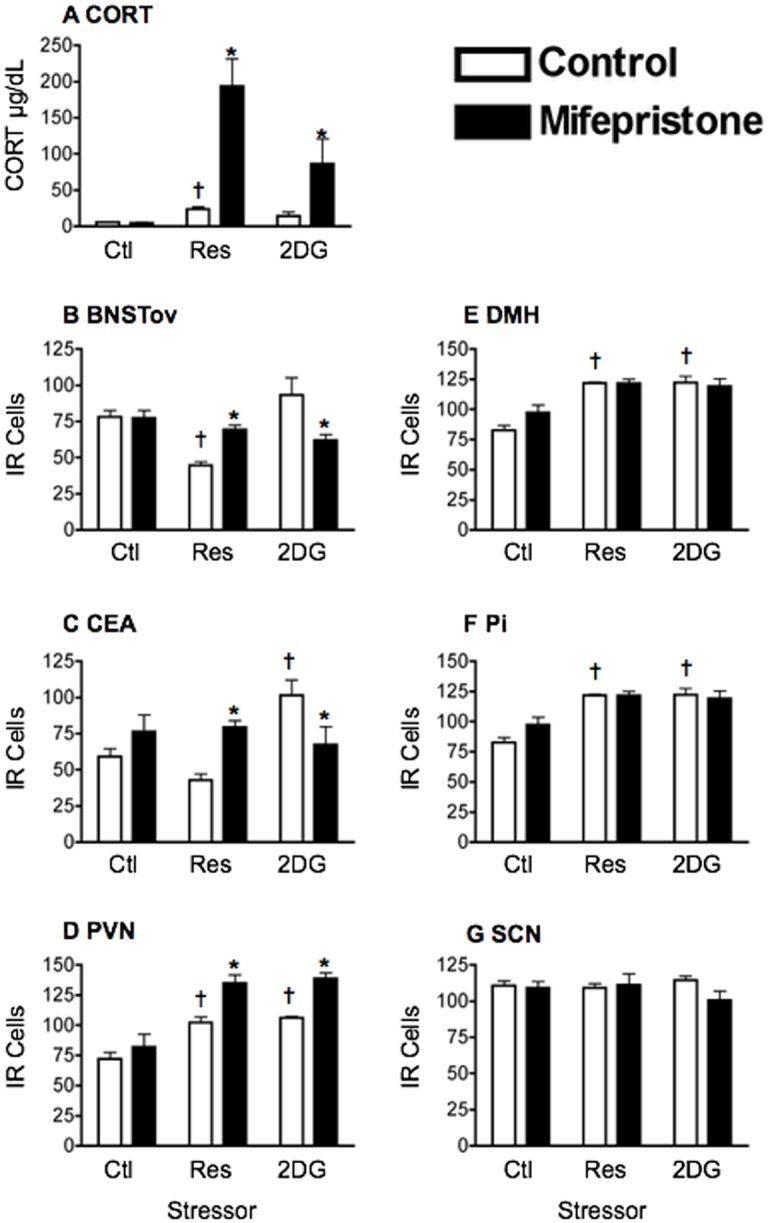
Effect of mifepristone on stress-induced changes in plasma CORT levels and PER1 expression. A) Plasma corticosterone (CORT) levels as a function of the category of stress and pharmacological blockade of glucocorticoid receptors. Rats were injected with saline (control) or mifepristone (GRX) at ZT1, 1 h prior to handling or stress exposure at ZT2. Plasma was collected 1 h later at ZT3. The bars and vertical lines represent means ± SEM, *n* = 4 per group. * Significant difference from corresponding control group (*p*<0.05). B-G) Number of PER1 immunoreactive (IR) cells in different forebrain and hypothalamic nuclei at ZT3. IR cells are shown as a function of category of stress in control and mifepristone-treated (GRX) rats. Means ± SEM are shown, *n* = 4 per group; * significant difference from corresponding control group, *p*<0.05. significant difference from main control group, *p*<0.05.

**Fig 4:**
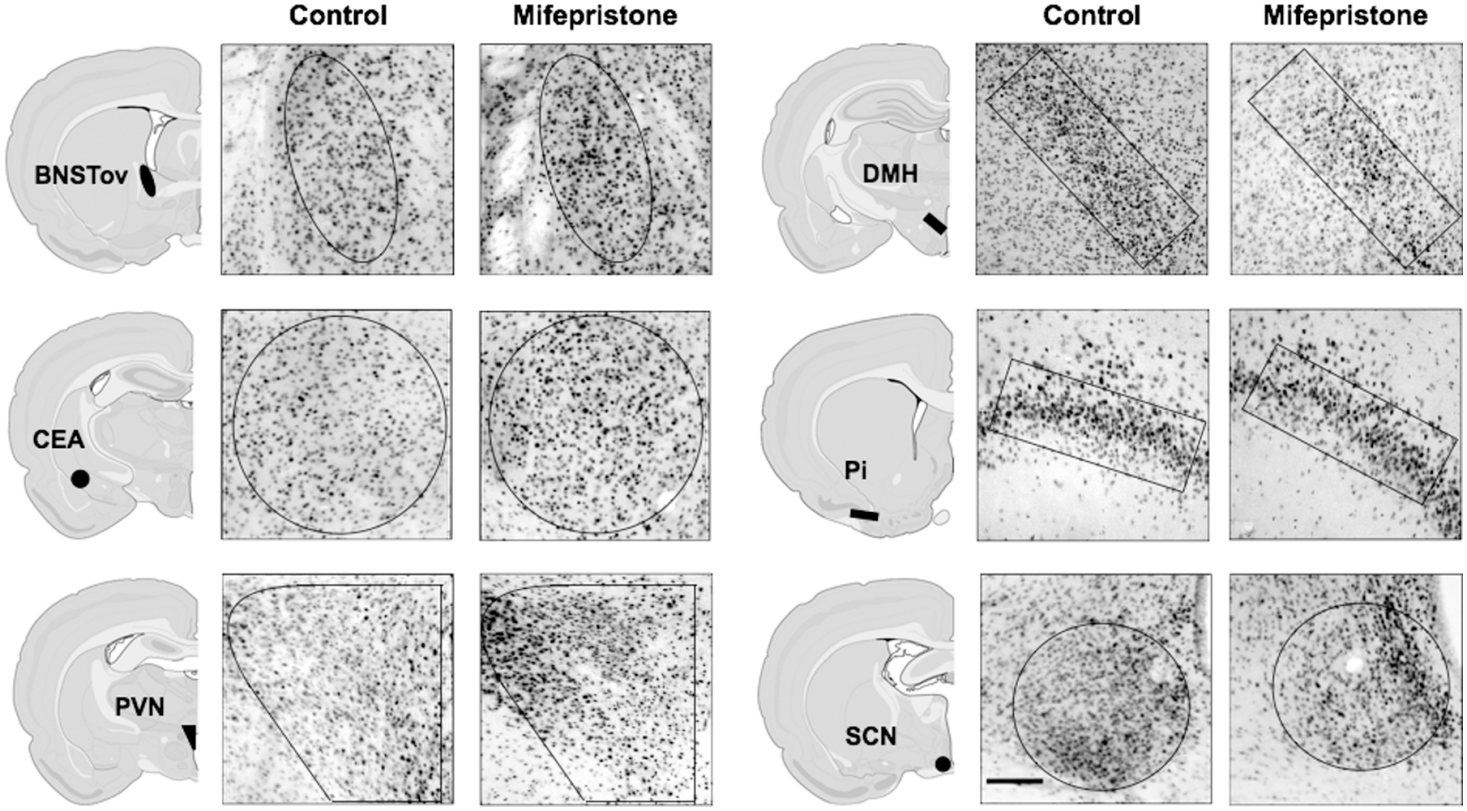
Examples of PER1 expression in different forebrain and hypothalamic nuclei of vehicle and mifepristone-treated rats. Rats were injected with vehicle or mifepristone at ZT1, exposed to a 30 min restraint session at ZT2, and then killed at ZT3 (scale bar: 100μm).

Plasma CORT levels in untreated rats increased following exposure to both restraint stress and 2DG (main effect of stress: F_2,17_ = 17.84, *p*<0.0001; Fig 3A). Treatment with the GR antagonist, mifepristone, had no effect on CORT levels in control group rats, but greatly enhanced the stress-induced circulating CORT levels (main effect of treatment: F_1,17_ = 34.75, *p*<0.0001; interaction: F_2,17_ = 12.15, *p*<0.001; Fig 3A). The effect of GR blockade on stress-induced PER1 expression is shown in Fig 3B–3G, Fig 4 and statistical analyses in Table 2. As with ADX, GR blockade prevented the restraint-induced suppression of PER1 expression in the BNSTov and CEAl, as well as the 2DG-induced increase in PER1 levels in these nuclei (Fig 3B and 3C). In addition, it attenuated the stimulatory effect of both stressors on PER1 levels in the DMH (Fig 3E). As with ADX, GR blockade accentuated the stress-induced increase in PER1 expression in the PVN (Fig 3D). Mifepristone treatment had no effect on the restraint or 2DG-induced increase in PER1 expression in the Pi (Fig 3F). PER1 levels in the SCN of control and mifepristone-treated animals were again unaffected (Fig 3G).

**Table 2:** Statistical analysis (ANOVA) of the effect of pharmacological blockade of glucocorticoid receptors (GRX) and stress type (Stressor) on PER1 expression in limbic and hypothalamic brain regions.

Structure	GRX	Stressor	GRX x Stressor
BNSTov	F_(1,18)_ = 0.22, ns	F_(2,18)_ = 7.79, *p*<0.01	F_(2,18)_ = 10.65, *p*<0.001
CEA	F_(1,18)_ = 0.84, ns	F_(2,18)_ = 3.62, *p*<0.05	F_(2,18)_ = 8.44, *p*<0.01
PVN	F_(1,18)_ = 24.78, *p*<0.0001	F_(2,18)_ = 33.05, *p*<0.0001	F_(2,18)_ = 2.26, ns
DMH	F_(1,17)_ = 39.89, *p*<0.0001	F_(2,17)_ = 7.55, *p*<0.01	F_(2,17)_ = 8.7, *p*<0.01
Pi	F_(1,18)_ = 0.97, ns	F_(2,18)_ = 27.41, *p*<0.0001	F_(2,18)_ = 1.95, ns
SCN	F_(1,17)_ = 1.12, ns	F_(2,17)_ = 0.16, ns	F_(2,17)_ = 1.34, ns

*ns* not significant

## Discussion

In mammals, the SCN clock governs the rhythmic secretion of glucocorticoid hormones [28]. In turn, glucocorticoids have been shown to play a role in the modulation of clock gene expression in subordinate clocks downstream of the SCN [7, 11, 29, 30]. In the present study we found, that depletion of endogenous glucocorticoids via ADX or treatment with the GR blocker, mifepristone altered the changes in PER1 expression induced by a neurogenic stressor, restraint, or by a systemic stressor, 2DG. These effects, however, were contingent upon the type of stress and brain region studied, where the central extended amygdala appears to be differentially sensitive to qualitative differences between systemic and processive stressors, as previously shown (21). This points to a complex role for glucocorticoid signaling in stress-induced changes in PER1 expression in the brain.

The attenuation of endogenous glucocorticoid signaling, via ADX or GR blockade, reversed the suppressive effect of restraint and also the stimulatory effect of 2DG on PER1 expression in the BNSTov and CEAl, regions that we have previously shown to exhibit rhythms of PER2 expression that are uniquely dependent on circadian glucocorticoid signaling. Specifically, we found that PER2 rhythms in these two regions were blunted by ADX [13, 14] and could be restored by giving CORT via the drinking water, a regimen that mimics endogenous CORT rhythms [7, 15, 31]. Furthermore, we found that the genetic deletion of GR restricted to brain tissues of mice blunted PER2 rhythms in the BNSTov and CEAl without affecting rhythms in other brain regions, including the SCN [31]. Thus, the present findings show that PER expression in the BNSTov and CEAl is sensitive not only to daily rhythms of glucocorticoid signaling, but also to acute stress-induced changes in circulating levels of glucocorticoid hormones. Our current work examines PER1 levels at only 1 time point, in the context of stress-induced protein expression almost immediately post-stress exposure. This notion builds upon our previous work (21) that explores the pleiotropic role of PER1 in regulating endogenous circadian oscillations, as well as perhaps the integrative arm of the stress system with that of the circadian one. In order to further address the former role and capture any modulatory effect of changes in glucocorticoids on local oscillations, it would be wise to assess stress-induced PER1, as well as PER2, levels through several time points across a 24 h cycle.

The neuronal subpopulations in which these glucocorticoid-mediated changes in PER1 occur remain to be determined. It has been previously established that systemic stressors increase *c-Fos* expression in GABA neurons containing enkephalin in the BNSTov and CEAl [32, 33], and that neurogenic stressor, such as restraint, suppress *c-Fos* in these nuclei [34]. In addition, glucocorticoids have been shown to modulate the expression of enkephalin in these regions [35, 36], suggesting that these are the neurons where stress and glucocorticoids hormones could interact to modulate PER expression. The fact, however, that the two different stressors, restraint, a neurogenic stressor, and 2DG, a systemic stressor, exert opposite effects on PER1 and also *c-Fos* expression in both regions [21, 34] clearly implies that other factors are interacting with glucocorticoids to mediate the effects of these different stressors on PER1 expression in BNSTov and CEAl.

Contrary to the opposite effects of the two stressors in the BNSTov and CEAl, in the DMH, both restraint and 2DG increased PER1 expression and these effects were prevented by ADX and by GR blockade, pointing to a permissive role for glucocorticoids on PER1 expression in this region. Curiously, in the PVN, a nucleus that receives major afferent inputs from the DMH [37–39], both ADX and GR blockade enhanced the effect of the two stressors on PER1 expression, pointing to an inhibitory role for glucocorticoids on PER1 in this region. Similar inhibitory effects of glucocorticoids on neural activity and on expression of different peptides (corticotropin-releasing hormone, adrenocorticotropic hormone, vasopressin, glutamate, gamma-aminobutyric acid) in the PVN have been previously reported [40–43]. Neither ADX nor GR blockade had any effect on stress-induced PER1 expression in the Pi, indicating that the regulation of PER1 expression in this region by stress is independent of glucocorticoid signaling. Finally, the lack of effects of either stressor or of glucocorticoid manipulations on PER1 levels in the SCN was to be expected and is consistent with previous evidence that the adult SCN is devoid of GR [7, 44], and unlike its downstream oscillators, is immune to the effects of glucocorticoids [11]. Taken together, this set of findings highlights an indirect role of stress-induced changes in circulating glucocorticoids in the regulation of clock gene expression in downstream oscillators of the forebrain.

Blockade of GR mimicked the effect of ADX, underscoring the importance of glucocorticoid signaling via GR in stress-induced changes in PER1 expression in the forebrain. The transient suppression of GR expression within select brain regions of interest, through dsRNA interference, can be used to further validate our findings and evade any consequences of global GR antagonism. There is some evidence that a GRE in the promoter region of the *Per1* gene mediates the effects of restraint stress on *Per1* transcription in mouse peripheral organs including the liver, kidneys and heart [9]. It is not known whether the *Per1* GRE is involved with any of the effects of stress on PER1 in the forebrain and, in particular, the suppressive effect of restraint in the BNSTov and CEAl. We found that acute changes in PER1 expression, resulting from a single stress exposure, were most robust in the short-term, 1 h post stress onset [21]. This suggests that stress-induced, glucocorticoid-dependent changes in brain PER1 expression may be mediated in parallel by rapid nongenomic mechanisms, perhaps via the activation of membrane-bound GR [41, 45, 46][41, 47].

In summary, the results of the present study show that acute exposure to categorically different stressors can induce rapid changes in PER1 expression, in a region-specific manner. Furthermore, these acute effects are attributed to changes in glucocorticoid signaling. It is possible that the stress-induced changes in PER1 can affect local clocks in the brain, leading to transient misalignment between the stress-resilient master clock and downstream brain oscillators, and ultimately to disruptions in circadian-controlled processes. Finally, it is important to note that PER1 has been implicated in behaviors such as stress-induced grooming, nociception, sensitization of drug effects, and anxiety-related behaviors in rodents [18, 20, 48–51], indicating that stress-induced changes in PER1 may have additional effects that are independent of the clock mechanism.

## References

[1] Albrecht U. Timing to perfection: the biology of central and peripheral circadian clocks. Neuron. 2012;74(2):246–60. Epub 2012/05/01. 10.1016/j.neuron.2012.04.006 . 22542179

[2] Mohawk JA, Green CB, Takahashi JS. Central and peripheral circadian clocks in mammals. Annual review of neuroscience. 2012;35:445–62. 10.1146/annurev-neuro-060909-153128 22483041 PMC3710582

[3] Wong CC, Schumann G. Integration of the circadian and stress systems: influence of neuropeptides and implications for alcohol consumption. Journal of neural transmission. 2012;119(10):1111–20. Epub 2012/06/01. 10.1007/s00702-012-0829-4 . 22648536

[4] Meerlo P, Sgoifo A, Turek FW. The effects of social defeat and other stressors on the expression of circadian rhythms. Stress. 2002;5(1):15–22. 10.1080/102538902900012323 . 12171763

[5] Weibel L, Maccari S, Van Reeth O. Circadian clock functioning is linked to acute stress reactivity in rats. Journal of biological rhythms. 2002;17(5):438–46. . 12375620 10.1177/074873002237138

[6] Pantazopoulos H, Dolatshad H, Davis FC. A fear-inducing odor alters PER2 and c-Fos expression in brain regions involved in fear memory. PloS one. 2011;6(5):e20658 10.1371/journal.pone.0020658 21655193 PMC3105109

[7] Segall LA, Amir S. Glucocorticoid regulation of clock gene expression in the mammalian limbic forebrain. Journal of molecular neuroscience: MN. 2010;42(2):168–75. Epub 2010/03/02. 10.1007/s12031-010-9341-1 . 20191328

[8] Takahashi S, Yokota S, Hara R, Kobayashi T, Akiyama M, Moriya T, et al Physical and inflammatory stressors elevate circadian clock gene mPer1 mRNA levels in the paraventricular nucleus of the mouse. Endocrinology. 2001;142(11):4910–7. Epub 2001/10/19. 10.1210/endo.142.11.8487 . 11606459

[9] Yamamoto T, Nakahata Y, Tanaka M, Yoshida M, Soma H, Shinohara K, et al Acute physical stress elevates mouse period1 mRNA expression in mouse peripheral tissues via a glucocorticoid-responsive element. The Journal of biological chemistry. 2005;280(51):42036–43. Epub 2005/10/27. 10.1074/jbc.M509600200 . 16249183

[10] Dickmeis T. Glucocorticoids and the circadian clock. The Journal of endocrinology. 2009;200(1):3–22. Epub 2008/10/31. 10.1677/JOE-08-0415 . 18971218

[11] Balsalobre A, Brown SA, Marcacci L, Tronche F, Kellendonk C, Reichardt HM, et al Resetting of circadian time in peripheral tissues by glucocorticoid signaling. Science. 2000;289(5488):2344–7. Epub 2000/09/29. . 11009419 10.1126/science.289.5488.2344

[12] Balsalobre A, Marcacci L, Schibler U. Multiple signaling pathways elicit circadian gene expression in cultured Rat-1 fibroblasts. Current biology: CB. 2000;10(20):1291–4. Epub 2000/11/09. . 11069111 10.1016/s0960-9822(00)00758-2

[13] Amir S, Lamont EW, Robinson B, Stewart J. A circadian rhythm in the expression of PERIOD2 protein reveals a novel SCN-controlled oscillator in the oval nucleus of the bed nucleus of the stria terminalis. The Journal of neuroscience: the official journal of the Society for Neuroscience. 2004;24(4):781–90. Epub 2004/01/30. 10.1523/JNEUROSCI.4488-03.2004 . 14749422 PMC6729822

[14] Lamont EW, Robinson B, Stewart J, Amir S. The central and basolateral nuclei of the amygdala exhibit opposite diurnal rhythms of expression of the clock protein Period2. Proceedings of the National Academy of Sciences of the United States of America. 2005;102(11):4180–4. Epub 2005/03/05. 10.1073/pnas.0500901102 15746242 PMC554834

[15] Segall LA, Perrin JS, Walker CD, Stewart J, Amir S. Glucocorticoid rhythms control the rhythm of expression of the clock protein, Period2, in oval nucleus of the bed nucleus of the stria terminalis and central nucleus of the amygdala in rats. Neuroscience. 2006;140(3):753–7. Epub 2006/05/09. 10.1016/j.neuroscience.2006.03.037 . 16678973

[16] Segall LA, Amir S. Exogenous corticosterone induces the expression of the clock protein, PERIOD2, in the oval nucleus of the bed nucleus of the stria terminalis and the central nucleus of the amygdala of adrenalectomized and intact rats. Journal of molecular neuroscience: MN. 2010;42(2):176–82. Epub 2010/04/28. 10.1007/s12031-010-9375-4 . 20422314

[17] Nader N, Chrousos GP, Kino T. Interactions of the circadian CLOCK system and the HPA axis. Trends in endocrinology and metabolism: TEM. 2010;21(5):277–86. Epub 2010/01/29. 10.1016/j.tem.2009.12.011 20106676 PMC2862789

[18] Spencer S, Falcon E, Kumar J, Krishnan V, Mukherjee S, Birnbaum SG, et al Circadian genes Period 1 and Period 2 in the nucleus accumbens regulate anxiety-related behavior. The European journal of neuroscience. 2013;37(2):242–50. Epub 2012/10/09. 10.1111/ejn.12010 23039899 PMC3711746

[19] Verwey M, Lam GY, Amir S. Circadian rhythms of PERIOD1 expression in the dorsomedial hypothalamic nucleus in the absence of entrained food-anticipatory activity rhythms in rats. The European journal of neuroscience. 2009;29(11):2217–22. Epub 2009/06/06. 10.1111/j.1460-9568.2009.06766.x . 19490091

[20] Zhang J, Wu Z, Zhou L, Li H, Teng H, Dai W, et al Deficiency of antinociception and excessive grooming induced by acute immobilization stress in Per1 mutant mice. PloS one. 2011;6(1):e16212 Epub 2011/01/26. 10.1371/journal.pone.0016212 21264262 PMC3021546

[21] Al-Safadi S, Al-Safadi A, Branchaud M, Rutherford S, Dayanandan A, Robinson B, et al Stress-Induced Changes in the Expression of the Clock Protein PERIOD1 in the Rat Limbic Forebrain and Hypothalamus: Role of Stress Type, Time of Day, and Predictability. PloS one. 2014;9(10):e111166 10.1371/journal.pone.0111166 25338089 PMC4206498

[22] Koresh O, Kozlovsky N, Kaplan Z, Zohar J, Matar MA, Cohen H. The long-term abnormalities in circadian expression of Period 1 and Period 2 genes in response to stress is normalized by agomelatine administered immediately after exposure. European neuropsychopharmacology: the journal of the European College of Neuropsychopharmacology. 2012;22(3):205–21. Epub 2011/09/20. 10.1016/j.euroneuro.2011.07.012 . 21925847

[23] Paladino N, Leone MJ, Plano SA, Golombek DA. Paying the circadian toll: the circadian response to LPS injection is dependent on the Toll-like receptor 4. Journal of neuroimmunology. 2010;225(1–2):62–7. Epub 2010/06/18. 10.1016/j.jneuroim.2010.04.015 . 20554031

[24] O'Callaghan EK, Anderson ST, Moynagh PN, Coogan AN. Long-lasting effects of sepsis on circadian rhythms in the mouse. PloS one. 2012;7(10):e47087 Epub 2012/10/17. 10.1371/journal.pone.0047087 23071720 PMC3469504

[25] Fuchs E, Flugge G. Chronic social stress: effects on limbic brain structures. Physiology & behavior. 2003;79(3):417–27. Epub 2003/09/05. . 12954436 10.1016/s0031-9384(03)00161-6

[26] Anisman H, Hayley S, Kelly O, Borowski T, Merali Z. Psychogenic, neurogenic, and systemic stressor effects on plasma corticosterone and behavior: mouse strain-dependent outcomes. Behavioral neuroscience. 2001;115(2):443–54. . 11345969

[27] Zalachoras I, Houtman R, Atucha E, Devos R, Tijssen AM, Hu P, et al Differential targeting of brain stress circuits with a selective glucocorticoid receptor modulator. Proceedings of the National Academy of Sciences of the United States of America. 2013;110(19):7910–5. Epub 2013/04/25. 10.1073/pnas.1219411110 23613579 PMC3651427

[28] Dickmeis T, Weger BD, Weger M. The circadian clock and glucocorticoids--interactions across many time scales. Molecular and cellular endocrinology. 2013;380(1–2):2–15. Epub 2013/05/28. 10.1016/j.mce.2013.05.012 . 23707790

[29] Kalsbeek A, van der Spek R, Lei J, Endert E, Buijs RM, Fliers E. Circadian rhythms in the hypothalamo-pituitary-adrenal (HPA) axis. Molecular and cellular endocrinology. 2012;349(1):20–9. Epub 2011/07/26. 10.1016/j.mce.2011.06.042 . 21782883

[30] Amir S, Robinson B. Thyroidectomy alters the daily pattern of expression of the clock protein, PER2, in the oval nucleus of the bed nucleus of the stria terminalis and central nucleus of the amygdala in rats. Neuroscience letters. 2006;407(3):254–7. Epub 2006/09/16. 10.1016/j.neulet.2006.08.057 . 16973268

[31] Segall LA, Milet A, Tronche F, Amir S. Brain glucocorticoid receptors are necessary for the rhythmic expression of the clock protein, PERIOD2, in the central extended amygdala in mice. Neuroscience letters. 2009;457(1):58–60. Epub 2009/05/12. 10.1016/j.neulet.2009.03.083 . 19429162

[32] Davis M, Shi C. The extended amygdala: are the central nucleus of the amygdala and the bed nucleus of the stria terminalis differentially involved in fear versus anxiety? Annals of the New York Academy of Sciences. 1999;877:281–91. Epub 1999/07/23. . 10415655 10.1111/j.1749-6632.1999.tb09273.x

[33] Kozicz T. Met-enkephalin immunoreactive neurons recruited by acute stress are innervated by axon terminals immunopositive for tyrosine hydroxylase and dopamine-alpha-hydroxylase in the anterolateral division of bed nuclei of the stria terminalis in the rat. The European journal of neuroscience. 2002;16(5):823–35. Epub 2002/10/10. . 12372018 10.1046/j.1460-9568.2002.02129.x

[34] Day HE, Nebel S, Sasse S, Campeau S. Inhibition of the central extended amygdala by loud noise and restraint stress. The European journal of neuroscience. 2005;21(2):441–54. Epub 2005/01/28. 10.1111/j.1460-9568.2005.03865.x 15673443 PMC2430886

[35] Pompei P, Riftina F, McEwen BS. Effect of adrenal steroids on preproneurokinin-A gene expression in discrete regions of the rat brain. Brain research Molecular brain research. 1995;33(2):209–16. Epub 1995/11/01. . 8750879 10.1016/0169-328x(95)00115-9

[36] Honkaniemi J, Pelto-Huikko M, Rechardt L, Isola J, Lammi A, Fuxe K, et al Colocalization of peptide and glucocorticoid receptor immunoreactivities in rat central amygdaloid nucleus. Neuroendocrinology. 1992;55(4):451–9. Epub 1992/04/01. . 1373477 10.1159/000126156

[37] Bailey TW, Dimicco JA. Chemical stimulation of the dorsomedial hypothalamus elevates plasma ACTH in conscious rats. American journal of physiology Regulatory, integrative and comparative physiology. 2001;280(1):R8–15. Epub 2000/12/22. . 11124128 10.1152/ajpregu.2001.280.1.R8

[38] Keim SR, Shekhar A. The effects of GABAA receptor blockade in the dorsomedial hypothalamic nucleus on corticotrophin (ACTH) and corticosterone secretion in male rats. Brain research. 1996;739(1–2):46–51. Epub 1996/11/11. . 8955923 10.1016/s0006-8993(96)00810-4

[39] Li HY, Sawchenko PE. Hypothalamic effector neurons and extended circuitries activated in "neurogenic" stress: a comparison of footshock effects exerted acutely, chronically, and in animals with controlled glucocorticoid levels. The Journal of comparative neurology. 1998;393(2):244–66. Epub 1998/04/21. . 9548700

[40] Tasker JG, Di S, Malcher-Lopes R. Rapid central corticosteroid effects: evidence for membrane glucocorticoid receptors in the brain. Integrative and comparative biology. 2005;45(4):665–71. Epub 2005/08/01. 10.1093/icb/45.4.665 . 21676815

[41] Tasker JG, Di S, Malcher-Lopes R. Minireview: rapid glucocorticoid signaling via membrane-associated receptors. Endocrinology. 2006;147(12):5549–56. Epub 2006/09/02. 10.1210/en.2006-0981 16946006 PMC3280589

[42] Weiser MJ, Osterlund C, Spencer RL. Inhibitory effects of corticosterone in the hypothalamic paraventricular nucleus (PVN) on stress-induced adrenocorticotrophic hormone secretion and gene expression in the PVN and anterior pituitary. Journal of neuroendocrinology. 2011;23(12):1231–40. Epub 2011/09/14. 10.1111/j.1365-2826.2011.02217.x 21910768 PMC3220769

[43] Di S, Malcher-Lopes R, Halmos KC, Tasker JG. Nongenomic glucocorticoid inhibition via endocannabinoid release in the hypothalamus: a fast feedback mechanism. The Journal of neuroscience: the official journal of the Society for Neuroscience. 2003;23(12):4850–7. Epub 2003/07/02. . 12832507 10.1523/JNEUROSCI.23-12-04850.2003 PMC6741208

[44] Akashi M, Nishida E. Involvement of the MAP kinase cascade in resetting of the mammalian circadian clock. Genes & development. 2000;14(6):645–9. Epub 2000/03/25. 10733524 PMC316464

[45] Strehl C, Gaber T, Lowenberg M, Hommes DW, Verhaar AP, Schellmann S, et al Origin and functional activity of the membrane-bound glucocorticoid receptor. Arthritis and rheumatism. 2011;63(12):3779–88. Epub 2011/09/08. 10.1002/art.30637 . 21898343

[46] Vernocchi S, Battello N, Schmitz S, Revets D, Billing AM, Turner JD, et al Membrane glucocorticoid receptor activation induces proteomic changes aligning with classical glucocorticoid effects. Molecular & cellular proteomics: MCP. 2013;12(7):1764–79. Epub 2013/01/24. 10.1074/mcp.M112.022947 23339905 PMC3708164

[47] Chen YZ, Qiu J. Possible genomic consequence of nongenomic action of glucocorticoids in neural cells. News in physiological sciences: an international journal of physiology produced jointly by the International Union of Physiological Sciences and the American Physiological Society. 2001;16:292–6. Epub 2001/11/24. . 11719608 10.1152/physiologyonline.2001.16.6.292

[48] Akhisaroglu M, Ahmed R, Kurtuncu M, Manev H, Uz T. Diurnal rhythms in cocaine sensitization and in Period1 levels are common across rodent species. Pharmacology, biochemistry, and behavior. 2004;79(1):37–42. Epub 2004/09/25. 10.1016/j.pbb.2004.06.014 . 15388282

[49] Dong L, Bilbao A, Laucht M, Henriksson R, Yakovleva T, Ridinger M, et al Effects of the circadian rhythm gene period 1 (per1) on psychosocial stress-induced alcohol drinking. The American journal of psychiatry. 2011;168(10):1090–8. Epub 2011/08/11. 10.1176/appi.ajp.2011.10111579 . 21828288

[50] Gery S, Komatsu N, Kawamata N, Miller CW, Desmond J, Virk RK, et al Epigenetic silencing of the candidate tumor suppressor gene Per1 in non-small cell lung cancer. Clinical cancer research: an official journal of the American Association for Cancer Research. 2007;13(5):1399–404. Epub 2007/03/03. 10.1158/1078-0432.CCR-06-1730 . 17332281

[51] Yang X, Wood PA, Ansell CM, Quiton DF, Oh EY, Du-Quiton J, et al The circadian clock gene Per1 suppresses cancer cell proliferation and tumor growth at specific times of day. Chronobiology international. 2009;26(7):1323–39. Epub 2009/11/18. 10.3109/07420520903431301 . 19916834

